# Editorial: Pandemic-driven telehealth uptake: the missing healthcare provider, system and patient voices

**DOI:** 10.3389/fdgth.2023.1293921

**Published:** 2023-10-16

**Authors:** Janet Michel, Mary Kawonga, Harvey Rubin

**Affiliations:** ^1^ Swiss Centre for International Health (SCIH), Swiss Tropical and Public Health Institute (Swiss TPH), Basel, Switzerland; ^2^ Swiss Centre for International Health (SCIH), University of Basel, Basel, Switzerland; ^3^ School of Public Health, University of the Witwatersrand, Johannesburg, South Africa; ^4^ Department of Medicine, School of Medicine, University of Pennsylvania, Philadelphia, PA, United States

**Keywords:** pandemic (COVID-19), telehealth, uptake, missing, patient voice, health care provider, system managers (SMs)

**Editorial on the Research Topic** Pandemic-driven telehealth uptake: the missing healthcare provider, system and patient voices

The COVID-19 pandemic catapulted health systems world-wide into telehealth, moving digital health from being an optional to an essential health system component. There was no time to assess what patients or the customer needed from digital health. Social distancing measures were put in place, and patients found themselves confronted and at times with telehealth as the only option. Health care providers and system managers scrambled to put telehealth systems to reduce the burden on the health system in place, when anxious, worried and sick patients flooded hospitals and emergency departments. There was no time to think through feasibility issues, neither was there time to test the tools for safety and efficacy.

The Frontiers in Digital Health Research Topic Pandemic-driven Telehealth Uptake: The Missing Healthcare Provider, System and Patient Voices was created to serve as a platform to explore, exchange and stimulate discussion on how these missing voices affected telehealth delivery and how they can be incorporated moving forward. The importance of inclusive co-design of digital interventions cannot be overemphasized (1). Co-designing health innovations with people with diverse expertise (e.g., lived-experience, clinical, research) improves the quality of the innovation and users' perceived acceptability and utility (2).

The editors of this Research Topic— Mary Kawonga of University of Witwatersrand, Harvey Rubin of University of Pennyslvania and Janet Michel from the Swiss Tropical and Public Health Institute hope that these articles will lead to a broader Global health discourse, looking into which elements of telehealth make sense and to whom, patients, health care provide or system managers? There are indications that patients want to see telehealth as an add on and not a replacement of face to face consultations (3). How do we optimize telehealth for patients to get the best at distance care and organizations to get the most out of their tech investment? (4).

The articles in this Research Topic, all a mix of systematic review and original research, exemplify creative inquiry into compelling problems within The Pandemic-driven Telehealth Uptake and The Missing Healthcare Provider, System and Patient Voices.

In the article, *An interactive national digital surveillance system to fight against COVID-19 in Bangladesh*, Sarker et al. described the design, development, implementation, and characteristics of a nationwide web-based surveillance system for educating, screening, and tracking COVID-19 at the community level in Bangladesh, using a mobile phone application and a cloud server, demonstrating the potential of telehealth in identifying patients at risk of COVID-19, referring them to the nearest government healthcare facility, tracking and tracing positive cases as well as documenting patient outcomes. This screening can help the symptomatic patient take immediate action, such as isolation or hospitalization, depending on the severity.

*Patients' choice preferences for specialist outpatient online consultations: A discrete choice experiment* by Wu et al. analyzed the choice preference of patients for specialist outpatient online consultations (SOOC) via a discrete choice experiment, revealing convenience as one of the influencing factors. Patients are more inclined to choose specialist outpatient online consultations when doctors highly recommend it, when it is convenient to apply and when disease severity is mild. The study showed differences in patient choice preference by age, whether the patients had chronic diseases, income, and medical insurance types.

*Switching from offline to online health consultation in the post-pandemic era: the role of perceived pandemic risk* by Pan et al. explored the users' perceptions towards both offline and online services, their intention to switch to OHC, and the perceived pandemic risks. The study provides novel insights into the understanding of OHC usage in the post-pandemic era, and also informs medical facilities, OHC platforms, and policymakers on managing and balancing the online and offline healthcare provision.

*Satisfaction with pediatric telehealth according to the opinions of children and adolescents during the COVID-19 pandemic: A literature review* by Kodjebacheva et al. reviewed satisfaction with telehealth among children and adolescents based on their own opinions during the COVID-19 pandemic. For children and adolescents, telehealth services received favorable satisfaction ratings most probably due to the convenience effect. The paediatricians, however seem to have a different view. The perceptions of paediatricians to telehealth were less positive as compared to child patents families (5, 6), hence the need to align the patient needs, health care provider and system capacities.

Participants expressed high satisfaction with video and telephone visits and home telemonitoring while also preferring a combination of in-person visits and telehealth services. Factors associated with higher satisfaction with telehealth included greater distance from the medical center, older age, and lower anxiety when using telehealth. In qualitative studies, preferred telehealth features among participants included: a stable Internet connection and anonymity and privacy during telehealth visits.

Like other industries, the health care sector is increasingly realising the transformative nature of IoT technologies, as advances in computing and processing power, wireless technology and miniaturisation drive innovation in connected medical device development. While the worst of it may be behind us, the pandemic yet lingers and what remains is the question: what do patients and healthcare providers wish to see in regard to telehealth-based care?

This Research Topic explored and highlight the telehealth needs of both patient and healthcare providers globally. It is imperative to explore telehealth needs, compare and contrast what both patients and providers expect from telehealth and bring the two in alignment. The needs of the customers need to drive telehealth. What can be digitalized. What cannot? The involvement and engagement of patients, health care and system providers, is essential. Exploratory qualitative research is needed to identify telehealth needs through comparing and contrasting what patients and health care providers expect and what systems managers see as feasible and where possible bring these in alignment. Lack of technological skills and resistance to change threaten to come in the way. Telehealth training for health care providers is needed. Some health care providers are not tech savvy. Lack of skills and resistance to change (attitudes) have been found to deter telehealth uptake. Training in digital competencies for health care providers is needed too. An additional challenge is scheduling, particularly when a provider caters for both online and face to face clients. Hybrid model can also entail that patient use an OFTT for history taking and then see a doctor afterwards. It is paramount to engage the health care providers and get to know what the health care providers expect from telehealth? What are the elements that need to be focused on in medical education? We were pushed into telehealth, now is the time to adapt and work on evaluating the impact of digital health. What can be digitalized. What cannot? The involvement and engagement of health care providers is essential. Coordinated, safe and cost-effective care is critical (7).

As key stakeholders, both patients and healthcare providers have an interest in being involved in and defining how digital healthcare will evolve moving forward, however there is no established framework for how this can be realistically achieved. This in our view is a gap that needs to be addressed. It is imperative to bear in mind that like any intervention, digital health produces both intended and unintended effects. Some suggest that digital health might not be beneficial to everyone, everywhere. As digital health and AI use in the health systems expand, it is therefore urgent to engage with all key stakeholders. What might benefit the health system might be a draw back for the patients and vice versa. Finding the means to balance these is more critical now than ever.
